# Linear fuzzy gene network models obtained from microarray data by exhaustive search

**DOI:** 10.1186/1471-2105-5-108

**Published:** 2004-08-10

**Authors:** Bahrad A Sokhansanj, J Patrick Fitch, Judy N Quong, Andrew A Quong

**Affiliations:** 1 Computational Systems Biology Group, Chemistry & Materials Science Directorate, University of California, Lawrence Livermore National Laboratory, L-235, 7000 East Ave., Livermore, CA, USA, 94551; 2 School of Biomedical Engineering, Science and Health Systems, Drexel University, Philadelphia, PA, USA; 3 Chemical & Biological National Security Program, University of California, Lawrence Livermore National Laboratory, Livermore, CA, USA; 4 Department of Oncology, Lombardi Cancer Center, Georgetown University Medical School, Washington, DC, USA

## Abstract

**Background:**

Recent technological advances in high-throughput data collection allow for experimental study of increasingly complex systems on the scale of the whole cellular genome and proteome. Gene network models are needed to interpret the resulting large and complex data sets. Rationally designed perturbations (e.g., gene knock-outs) can be used to iteratively refine hypothetical models, suggesting an approach for high-throughput biological system analysis. We introduce an approach to gene network modeling based on a scalable linear variant of fuzzy logic: a framework with greater resolution than Boolean logic models, but which, while still semi-quantitative, does not require the precise parameter measurement needed for chemical kinetics-based modeling.

**Results:**

We demonstrated our approach with exhaustive search for fuzzy gene interaction models that best fit transcription measurements by microarray of twelve selected genes regulating the yeast cell cycle. Applying an efficient, universally applicable data normalization and fuzzification scheme, the search converged to a small number of models that individually predict experimental data within an error tolerance. Because only gene transcription levels are used to develop the models, they include both direct and indirect regulation of genes.

**Conclusion:**

Biological relationships in the best-fitting fuzzy gene network models successfully recover direct and indirect interactions predicted from previous knowledge to result in transcriptional correlation. Fuzzy models fit on one yeast cell cycle data set robustly predict another experimental data set for the same system. Linear fuzzy gene networks and exhaustive rule search are the first steps towards a framework for an integrated modeling and experiment approach to high-throughput "reverse engineering" of complex biological systems.

## Background

While similarity (homology) of DNA sequence between organisms can be used to propose *potential *gene functions, transcriptional regulation, and protein pathways (e.g., [1]), there are often major differences in the protein products, functions, and pathway involvement of genes with nearly identical sequences [2]. Consequently, sequence homology may be viewed as a means of generating an initial "draft" hypothesis for the gene network of a newly sequenced organism that can be built upon using high throughput experimental techniques such as DNA chips and microarrays for mRNA transcript profiling [3], protein abundance profiling with mass spectroscopy and 2-D gel electrophoresis [4], and protein-protein and protein-DNA binding assayed using SELDI mass spectrometry [5] and protein chips [6]. In addition, new genetic technologies, in particular small interfering RNA (siRNA) for selective gene suppression facilitate high-throughput massively parallel perturbation of the gene and protein networks of biological systems [7].

Given the potential scale and complexity of experiments and resulting data sets, biologists need a modeling and simulation framework to optimally design experiments and interpret results. The problem is not simply one of "reverse engineering" to find the optimal "best fit" gene, protein, and/or metabolite interaction model to explain a set of experimental results; rather, modeling should suggest the range of hypotheses that can potentially explain the results of one experiment and select the optimal next experiment to reduce the number of possible alternative hypotheses, with the goal of converging to a biological system model that can be used to predict the effect of molecular perturbations.

A major challenge of modeling biological systems is that conventional methods based on physical and chemical principles require data that is difficult to accurately and consistently obtain using either conventional biochemical or high throughput technologies, which typically yield noisy, semi-quantitative data (often in terms of a ratio rather than a physical quantity) [3]. In particular, microarray gene expression ratios are ultimately obtained from pixel counts of relatively messy images. Boolean networks (e.g. [8]) are computationally simple and do not depend on precise experimental data, and thus they are suitable for handling both the complexity of biological networks and the challenge of generating and comparing multiple hypothetical networks as described in the above scheme. However, Boolean models have inadequate dynamic resolution to accurately describe the behavior of a biological network [9]. In contrast, differential equation models (e.g., [10]) can be computationally expensive and sensitive to imprecisely measured parameters. Even the lower throughput RT-PCR method for gene expression measurement (as described in [10]) cannot produce quantitatively precise data that can be accurately mapped to actual mRNA concentrations in the sample. Because of computational limitations, continuous modeling approaches (e.g., [10,11]) are limited to finding the single model that best fits experimental data given some set of constraints, such as a maximally sparse gene interaction network [11].

Fuzzy logic [12] provides a mathematical framework that is compatible with poorly quantitative yet qualitatively significant data. Fuzzy logic is a natural language for linguistic modeling, thus it is consistent with the qualitative linguistic-graphical methods conventionally used to describe biological systems. Fuzzy models are rule-based; accordingly, there is a potential scalability problem as the number of antecedents ("inputs" to the rule) and variable states ("resolution" of inputs and rule outputs) increase, causing combinatorial explosion. Non-scalable conventional fuzzy logic has previously been used to analyze microarray data [13]. However, because of the nonlinear scalability of the modeling method and resulting computational expense of generating rules for multiple inputs, this method allows for only one possible positive and one possible negative regulator for each gene, thus yielding few biologically meaningful insights and experimentally testable hypotheses.

The problem of rule set combinatorial explosion is addressed by the union rule configuration (URC) developed by Combs and Andrew [14], which allows for linear growth in rule set complexity with both resolution (number of states) and number of inputs (rule antecedents) at the cost of having to represent nonlinear relationships between inputs as hidden layers [15]. In the linear (URC) fuzzy logic scheme, there are distinct fuzzy rules for each individual input to a given output. For example, given input variables A and B to an output C, there would be a set of rules relating A to C (e.g., "If A is LOW then C is LOW", "If A is HIGH then C is HIGH") and another set of rules relating B to C (e.g. "If B is LOW then C is HIGH", "If B is HIGH then C is LOW"). After each rule is applied individually, the intermediate evaluations of the fuzzy state of the output variable ("node") are aggregated by a fuzzy union (logical OR) operation (e.g. by summing or taking the resulting memberships in the fuzzy sets defining the state of the output). This contrasts with conventional fuzzy logic (or the "intersection rule configuration"), which has rules relating all combinations of inputs evaluated by a fuzzy intersection (logical AND). For the example with inputs A and B to output C, rules would read as, e.g., "If A is LOW and B is LOW then C is LOW", "If A is LOW and B is HIGH then C is HIGH", etc., leading to a combinatorial explosion avoided by the URC.

The utility of linear (URC) fuzzy logic has been demonstrated in its ability to qualitatively model the *lac *operon of *E. coli *[16,17]. In our previous work, a URC fuzzy logic model was constructed from existing qualitative biological knowledge about the interaction of genes and limited quantitative data on protein and metabolite concentration and enzyme kinetics, showing the power of linear fuzzy logic to describe complex multi-component regulation. Here, the linear fuzzy logic method is extended to tackle the inverse problem of gene network reconstruction from real quantitative microarray data where there are many inputs. This involves both methods for mapping the experimental data to the fuzzy logic membership functions and a useful implementation of the URC fuzzy logic to represent the gene networks. In addition, a robust algorithm for performing searches through the exponentially large space of possible gene networks is presented.

To address the problem of generating *all *plausible hypothetical network models that explain an experimental data set, we are initially proceeding with an exhaustive search of possible gene interactions to find those that fit the data within some error threshold. Thus, the problem we are tackling is of exponential complexity with O(*m*^*N*^) growth in the number of possible rules for the behavior of a given "output" gene of a gene interaction node, where *N *is the number of (input) genes that can possibly control it and *m *is the number of possible rules describing the effect of each single input gene on the output gene. On the other hand, if a linear fuzzy logic scheme is not used, the problem would grow at an unacceptably high O(*m*^*N^N*^) rate. The number of possible rules for each gene-gene interaction (*m*) is given by *n*^*n*^, where *n *is number of fuzzy sets that describe the state of a variable. Hence, we will constrain the size of the problem by (i) setting the minimum number of fuzzy sets to three, the minimum for meaningful resolution, (ii) limiting the number of possible input genes that are allowed to control the output of the output gene at each node of the fuzzy network model, and (iii) not allowing nonlinear gene interactions which would require hidden layers. The last condition is not particularly severe, as a typical nonlinear interaction (e.g., "xor") interaction between two regulatory proteins at a gene is mediated by an intermediate complex between the proteins that can be represented as an independent node in a network model. Therefore, "hidden layers" may generally be avoided by including more biological detail as explicit nodes in the model: for example, explicitly including the temporary interaction between proteins within a scaffolded cellular signal transduction complex, or by incorporating as model nodes the various topological states of a region of DNA influencing transcription factor binding, or in general, adding sufficient biological detail such that interactions between inputs can be linearly modeled.

We apply partially scalable, linear fuzzy network modeling to a data set commonly used for demonstrating computational methods in systems biology, microarray experiments of yeast cell cycle gene expression [18]. These data were obtained in 1998, prior to subsequent technical and statistical advances to improve data quality. However, to keep our case study as general as possible and demonstrate the ability of the fuzzy logic approach to handle other similarly noisy data sets, we do not do any data processing other than the fuzzy modeling process (described in the Methods). Exhaustive search is used as a brute force "reverse engineering" method to find all possible gene network models that fit the data for a set of twelve genes known to participate in the yeast cell cycle.

We show that the search converges to a small number of models describing the expression of each gene within a fit tolerance. Models found from the data for one particular yeast cell cycle time series are also capable of qualitatively predicting data from another time series experiment (i.e., one using a different cell synchronization method). In addition, given the constraints of the search algorithm (described in more detail in the Methods) and our limitation to pure transcriptional data, we find that the best fitting fuzzy network models collectively recover some direct and indirect functional relationships between genes predicted by interactions found by previous biochemical experiments as well as quantitative and statistical methods based on transcriptional correlations.

## Results

### Yeast cell cycle data set

As a proof of concept, we have used exhaustive search to generate fuzzy gene networks based on yeast (*Saccharomyces cerevisiae*) cell cycle microarray time series data sets presented in [18] (which included data from [19]). Researchers frequently use these data sets to demonstrate and validate statistical and clustering analysis (e.g., [20,21]), mathematical modeling [22,23], and reverse engineering methods [21,24]. Biological details of the yeast cell cycle transcriptional network and some computational methods for its analysis are reviewed in [25].

*S. cerevisiae *cell cycle regulatory protein-DNA interactions were also the subject of a recent extensive experimental study [26] and there is a large amount of previously obtained biological knowledge on the interaction of yeast cell cycle proteins, i.e., information contained in the Yeast Proteome Database [27] and the KEGG pathway database [28]. Consequently, predicted transcriptional network models we derive for the Spellman *et al*. [18] data set can be tested against numerous independent data sets and compared with models obtained using other methods.

**Figure 1:**
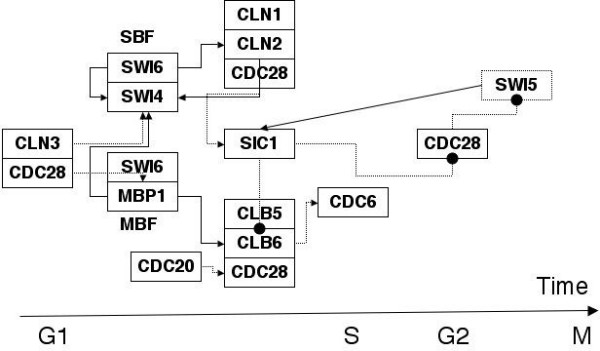
**Known Yeast Cell Cycle Interactions. **Schematic of known yeast cell cycle interactions between protein products of the twelve genes we studied (Table 1). An arrow indicates a positive interaction and a closed circle indicates a negative interaction. A dashed line indicates a protein-protein (e.g., phosphorylation, etc.) interaction and solid lines indicate direct transcriptional regulation. Genes are shown adjacent to each other when they are treated as a cluster in the KEGG pathway diagram (in the case of SBF and MBF the genes code for protein subunits). A connection inside a cluster indicates regulation of the whole cluster simultaneously. The interactions are located along an approximate time axis through the stages of the cell cycle (G1, S, G2).

We focus on the 12 key yeast cell cycle genes listed in Table 1 with descriptions taken from the Yeast Proteome Database. The protein products of these genes have been extensively studied using conventional biological techniques and are known to regulate each other and play key roles in controlling cell cycle. Consequently, observed correlations between the genes of Table 1 in cell cycle microarray data are most likely the result of real biological activity rather than noise. In addition, cell cycle gene subsets similar to this one have been the subject of other recent gene network modeling and reverse engineering publications (e.g., [21,24]). Figure 1 shows the current understanding of the interactions of the cell cycle protein subset.

There are three sets of gene expression time series in [18] measured for cells synchronized by different methods, called the *cdc15*, *alpha*, and *cdc28 *sets. We fit models on the basis of the *cdc15 *data set since it contains the least number of missing data. Time points in the *cdc15 *set for which there is missing data for one or more of the 12 genes are excluded from the rule search. We perform an exhaustive search with a maximum of 4 inputs per node, as detailed in the Methods. A Microsoft Excel workbook with the complete fitting data set is provided in Additional File 1, including all the fuzzy rule models for each gene obtained from exhaustive search with an *E*_MIN _threshold of approximately 0.6.

### Results of fitting to data

**Figure 2:**
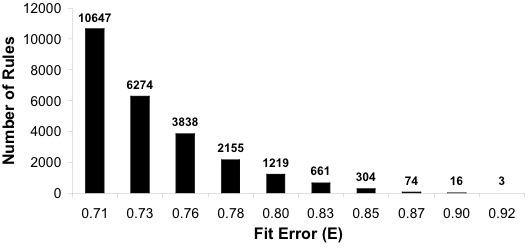
**Fuzzy Rule Search Convergence. **Histogram of the number of fuzzy rules for *CLN1* expression at different fit error (*E*) levels.

Figure 2 shows the number of rule models found in the exhaustive search that fit the expression time series of the *CLN1 *gene (using the *cdc15 *data set) at different error tolerance levels (*E*_MIN_, as defined by Equation 4 in the Methods). It shows typical behavior for the exhaustive rule search. The number of fuzzy models that fit a gene expression time series decreases exponentially as the fit tolerance (*E*_MIN_) increases, up to a maximum tolerance above which no models fit the data. A successful search generally converges to a small number of distinct models at the maximum fit tolerance, representing "plausible" hypothetical transcriptional networks that can explain the available data. In some cases, though it did not occur for any of the genes analyzed here, the search fails and there are a large number of models with similar poor fit scores and no suitable subset of "plausible" models.

**Figure 3:**
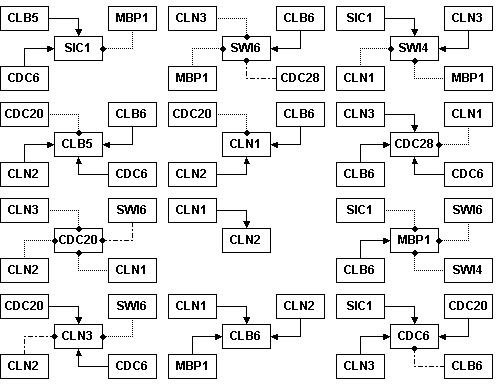
**Best Fit Model of Yeast Cell Cycle Interactions. **Diagram of the best fitting gene interactions for each node gene, as shown in Table 3. Solid lines ending in an arrow represent positive interactions (induction, e.g., [1 2 3], [1 3 3], etc.), dotted lines ending in a closed circle represent monotonic negative interaction (repression, e.g., [3 2 1], [3 1 1], [1 1 2], etc.), and dash-dot lines ending in a diamond represent an interaction that is both positive and negative (e.g., [3 1 3], [1 3 1], etc.).

The plausible model subset generally contains common rule patterns. For example, Table 2 lists the models for *CLB5 *expression with the highest fit scores found in the exhaustive search. The rules are in the format used for the example described in the Methods section. Table 3 shows three models for each gene in the network: the best fitting rule (highest score) and the two highest scoring rules with different combinations of input genes. The scores for each of the three models are provided in corresponding rows at the bottom of the table. Figure 3 shows the best fitting interaction network diagrams for each node gene from Table 3.

**Figure 4:**
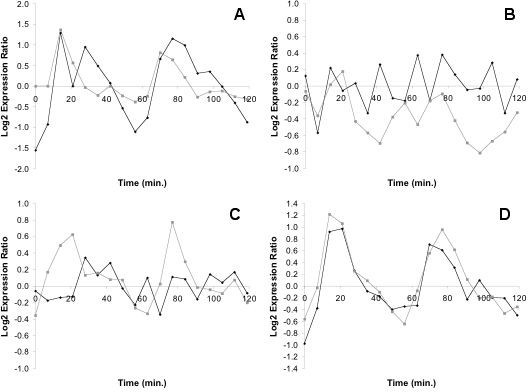
**Fuzzy Model Fit to Numerical Data. **Comparison of experimental (solid black line) gene expression ratios from the *alpha *cell synchronization data set (Spellman, *et al*., 1998) and the predicted time series of the best-fitting fuzzy model (grey line) for genes *CLN1*, *E *= 0.930 (**A**), *CDC28*, *E *= 0.510 (**B**), *SWI6*, *E *= 0.620 (**C**), and *CLB5*, *E *= 0.881 (**D**).

To test whether the linear fuzzy gene network models found for one set of experimental data (i.e., *cdc15 *synchronization time series) can accurately predict another set of results for the same system, we analyzed the microarray time series for *alpha *cell synchronization presented in [18]. There are some missing values for some genes at some time points in the *alpha *data set, which are set to zero and could potentially lead to discrepancies between the modeling and experimental data only at those points. Figure 4 shows the predicted time series for the expression ratio of four genes (*CLN1*, *CDC28*, *SWI6*, and *CLB5*) given the highest (except for *CLN1*, second-highest) scoring models in Table 3. (The second-best fitting model is used for *CLN1 *because it consists of four inputs, including all three inputs in the highest-scoring model along with another gene. Thus, it represents a more general "consensus" model for *CLN1*.) These models fit the original *cdc15 *training data with very different calculated tolerances (as measured by the fit error *E*) ranging from 0.510 (*CDC28*, Figure 4B) to 0.930 (*CLN1*, Figure 4A).

## Discussion

Using exhaustive search, we found linear fuzzy networks that predict *cdc15 *cell cycle microarray data for the expression of most of the twelve yeast genes we analyzed. The rule search typically converged to a small set of "plausible" models at a given fit error (*E*) tolerance for each gene (with exponential convergence as shown in Figure 2). Even for genes for which no highly fitting model could be found, such as *SWI4*, the best model (fitting at *E *= 0.620) predicts the qualitative behavior of independently measured *alpha *time series data (Figure 4C). Moreover, models that are more predictive (*E *> 0.8) of the *cdc15 *training data provide quantitatively accurate predictions of the *alpha *data (Figures 4A and 4D). Notably, these consistently good fits for the *alpha *data set were achieved using exactly the same arctangent data normalization and fuzzification scheme applied to the *cdc15 *data set. This suggests that the fuzzy processing methods described here can be generally applied for data sets obtained from different microarray experiments, provided a roughly symmetric distribution of Log2 ratios about 0, such that sets **1 **and **3 **both remain meaningful – though the ratios could be re-centered if necessary. In general, our results demonstrate the ability of qualitative fuzzy rule models to interpret the results of quantitative data and make predictions that can be statistically analyzed. Consequently, these models can be used to pose experimentally testable hypotheses.

Measurements of mRNA expression from microarray experiments complement information from additional gene knockout, DNA-protein and protein-protein experiments. A model based on pure transcriptional data will thus necessarily contain indirect relationships between proteins and miss other direct purely protein-protein interactions. However, gene network models can suggest functional roles and relationships for genes and proteins, and these models are necessary in complex system analysis to design and interpret further experiments that will specifically determine protein function and identify actual chemical interactions. To see what biological insights can be derived from fuzzy gene network models, we can examine areas of agreement and discrepancies between the best-fitting models found in our exhaustive search (shown in Table 3 and Figure 3) and the current understanding of the yeast cell cycle network (summarized in Figure 1).

Focusing on *CLN1*, we found positive regulation by *CLN2 *and negative regulation by *CDC20 *(Figure 3), which are correlations expected from biological knowledge (as shown in Figure 1) and found by Soinov, *et al*. using a supervised learning method [21]. In addition, the model for *CLN1 *includes a direct connection with *CDC28 *and an indirect connection with *MBP1 *(through regulation of the SBF complex) that are consistent with their relative positions in the cell cycle (Figure 1). The best-fit model for *CLN1 *depended solely on a positive interaction with *CLN2*, revealing the strong co-transcriptional connection between *CLN1 *and *CLN2*. The connection between *CLB5 *and *CLB6 *was also found in the model for *CLB5*. Other successfully found interactions include the negative regulation of *CDC6 *by *CLB6 *and the positive regulation of *CLB6 *by *MBP1*.

Some biologically accurate relationships were found that were absent from the supervised learning analysis of [21]. Notably, the model successfully recovers the apparent inhibition of *CLB5 *by *CDC20*, which is not shown in Figure 1 (based on the KEGG pathway) but arises from cdc20 protein presenting clb5 protein to proteases for degradation (as included in the model of [22], references within). There are several biological relationships that are not found in the best-fitting networks of Figure 3, such as an interaction between *SWI6 *and *SWI4 *(which form a multiprotein complex). The best-fitting models for *SWI4 *include a repressing action by *MBP1*, which is inconsistent with biological knowledge (Figure 1) suggesting that *MBP1 *and *SWI4 *activity should correlate (since they act at the same stage in the cell cycle). However, closer examination of the Spellman data set reveals that the amplitude of *MBP1* transcription varied within a small range, and the measurement could have been very noisy, resulting in a potential error by the algorithm. (It should be noted that no correlation is identified between *MBP1 *and *SWI4 *by the supervised learning algorithm in [21].)

In general, determining which relationships found in the fuzzy gene network represent biologically accurate interactions is a question that must be resolved by analyzing other data sets or from new experiments. The multiple plausible hypothetical input gene combinations can be used to optimally design experiments to add most information for least effort (time and cost) to revise fit errors and produce a new, more realistic set of hypothetical networks.

## Conclusions

In this work, we describe partially scalable, linear fuzzy logic models for biological network modeling. We demonstrate our approach by developing network models that accurately predict transcriptional data from typically noisy and semi-quantitative microarray experiments. Looking at the transcription network alone provides us with a view of the system at the "gene interactions" level. As measurement technology rapidly advances, the methods we describe can be extended to comprehensive heterogeneous data sets. To address the problem of analyzing the complex results of an exhaustive fuzzy model search and designing optimal experiments, we are currently developing pattern recognition methods to better visualize and interpret potentially large sets of models. In addition, we are considering stochastic methods to accurately sample and characterize the "space" of all possible fuzzy models to (a) more efficiently identify the subset of plausible models and (b) identify common patterns among all the models to gain a better understanding of the system and its evolution. While it is tempting to develop methods to obtain a single "optimal solution" as in a classic inverse problem, this is not appropriate for complex biological systems. Scarcity of both data and biological understanding mean that at best experiments will merely limit the space of potential solutions.

Biological system analysis is a *dynamic *reverse engineering problem, requiring continuous acquisition of new experimental data – data that should be acquired from experiments designed and informed by continuous modeling. Linear fuzzy rule network models are a promising methodology for an integrated modeling and experimental approach. Since fuzzy rule models are enumerable, methods developed for combinatorial optimization can be extended to them. Moreover, linear fuzzy network models can simultaneously contain both quantitative and qualitative information, providing a common framework for a broad range of biological data, including mass spectrometry analysis, RT-PCR, single cell imaging, metabolite profiling, and other technologies yet to be developed.

## Methods

### Converting between numerical data and fuzzy sets

**Figure 5:**
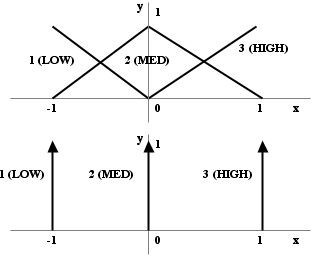
**Numerical-Fuzzy Data Conversion. **Fuzzification (top) and defuzzification (bottom) schemes used in this analysis, showing conversion to and from values in the interval [-1,1] on the x-axis and fuzzy sets LOW (**1**), MED (**2**), and HIGH (**3**). See text for details.

We use three fuzzy sets, Low (or **1**), Medium (**2**), and High (**3**) to represent the magnitude of gene expression, as defined in Figure 5. *Fuzzification *(conversion to fuzzy representation) of a numerical datum *x *is performed by finding the corresponding fuzzy set memberships *y*_1_, *y*_2_, and *y*_3 _(with values ranging from 0 to 1.0) given the linear functions shown in Figure 5, where


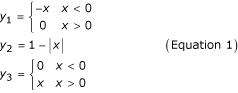


*Defuzzification *(conversion back to numerical representation) is performed using the "simplified centroid method" [29], with point set definitions shown in Figure 5. Following a fuzzy rule evaluation that returns fuzzy set memberships **y **= [*y*_1_ *y*_2_ *y*_3_] in sets **1 **(Low), **2 **(Med), and **3 **(High) respectively, the estimated numerical result of the evaluation, 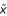, is given by the centroid for the points located at -1, 0, and +1 for each set respectively, or


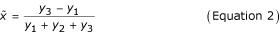


The fuzzy set definition and centroid defuzzification of Equations 1 and 2 were selected to maximize computational efficiency during exhaustive search: all 27 rules can be represented by easily implemented algebraic functions and it is possible to design the implementation to avoid as many costly if/then comparisons as possible. In addition, the scheme perfectly reproduces monotonic linear positive and negative interactions (i.e., the functions *f*(*x*) = *x *and *f*(*x*) = -*x *are quantitatively equal to monotonic fuzzy rules, which can be written using notation from the following section as [1 2 3] and [3 2 1] respectively) so it generally will not introduce systematic error in the model.

To apply this scheme for defuzzification and fuzzification scheme, experimental data must be projected on to the interval -1.0 through +1.0. Thus, log base 2 expression ratios are normalized by taking the arctangent of each ratio and dividing by π/2, yielding a symmetric transformation covering the desired interval. Previous work normalized expression ratios by the maximum value found in the experiment [17] or used different fuzzy set definitions for each variable [16], but those approaches suffer from a lack of universality across data sets and makes it difficult to compare and integrate data from different experiments. On the other hand, the arctangent method is defined across infinity, so no data will be "out of range". It also takes into account the fact that gene expression ratios often "saturate", and the difference between different degrees of high and low ratios are not necessarily biologically significant (this is because of the optical methods for measuring microarrays and the exponential error introduced using RT-PCR). When used in conjunction with the overlapping fuzzy set mappings shown in Figure 5, these "middle" values will tend to land in the Medium set (**2**).

### Comparing fuzzy predictions to numerical data

The fuzzy rule relating the input of a single gene to an ouptut node gene can be expressed as a rule vector **r**. For example, the rule **r **= [3 2 1] corresponds to the linguistic rules:

If *Input *is Low (**1**) then *Output *is High (**3**)

If *Input *is Med (**2**) then *Output *is Med (**2**)

If *Input *is High (**3**) then *Output *is Low (**1**)

Given the fuzzified expression of an input gene **y **= [*y*_1_ *y*_2_ *y*_3_] obtained using Equation 1 and the general fuzzy rule **r **= [*r*_1_ *r*_2_ *r*_3_], the resulting fuzzified expression of the output gene **z **will be:


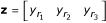


In general, node behavior is the result of *N *input genes acting on the output gene simultaneously. In the linear fuzzy logic scheme, the rule for each input gene is evaluated separately, leading to intermediate outputs **z**^*i*^:


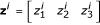


These intermediate fuzzy values are summed algebraically to obtain the final resulting fuzzy value for node gene expression:


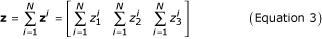


This result is defuzzified using Equation 2 to evaluate the output of the node. For three fuzzy sets, there are 3^3 ^or 27 possible rules describing the effect of a single gene on another gene. Thus, if there are *N *input genes for a node, there are 27^*N *^total possible rule combinations describing the behavior of the node gene.

In general, no rule combination will be an exact fit to real experimental data. Given some tolerance to fitting error, there will be multiple possible rule combinations, representing plausible hypothetical gene network models. In our present work, we define the error of the fit for the *M *data of the output gene **x **= {*x*_1_,*x*_2_,...,*x*_*M*_} as


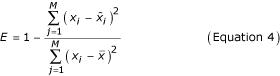


where 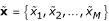 is the set of defuzzified numerical predictions (typically log expression ratios) and 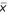 is the mean of the experimental data set **x**. A perfect fit results in a maximum *E *of 1.0. This error score was chosen because while it is quantitative, it emphasizes the correlation in qualitative behavior between the fit and prediction instead of the absolute numerical fit, which can be difficult to model with the limited resolution of three fuzzy sets. We can use the fitting error to rank these models, and use rule patterns consistent throughout all plausible models as a basis for constructing the template of a final network model that can be tested experimentally.

### Example of fuzzy rule evaluation

As an example to illustrate fuzzy gene networks using a simple rule combination, we consider three genes (*G1*, *G2*, *G3*) with log base 2 expression ratios measured at three different times:

*G*1 = {-3.0 0 +3.0}

*G*2 = {0.3 0 -0.3}

*G*3 = {+1 0 -1.0}

Using the arctangent normalization to project the ratios on [-1,1], we obtain

*G*1 = {-0.795 0 +0.795}

*G*2 = {+0.186 0 -0.186}

*G*3 = {+0.500 0 -0.500}

which can be fuzzified using Equation 1 to yield:

*G*1 = {[0.795 0.205 0] [0 1.0 0] [0 0.205 0.795]}

*G*2 = {[0 0.814 0.186] [0 1.0 0] [0.186 0.814 0]}

*G*3 = {[0 0.5 0.5] [0 1.0 0] [0.5 0.5 0]}

with vectors for each time point containing set membership in Low (**1**), Medium (**2**), and High (**3**). Consider the following rules for *G1 *and *G2 *as input genes to *G3*:

*G*1:*G*3 = [3 2 1]

*G*2:*G*3 = [1 2 3]

where the rules can be written in English as

If *G1 *is Low (**1**) then *G3 *is High (**3**)

If *G1 *is Med (**2**) then *G3 *is Med (**2**)

If *G1 *is High (**3**) then *G3 *is Low (**1**)

If *G2 *is Low (**1**) then *G3 *is Low (**1**)

If *G2 *is Med (**2**) then *G3 *is Med (**2**)

If *G2 *is High (**3**) then *G3 *is High (**3**)

Now, the evaluations of the rules taken individually are

*G*1:*G*3 = {[0 0.205 0.795] [0 1.0 0] [0.795 0.205 0]}

*G*2:*G*3 = {[0 0.814 0.186] [0 1.0 0] [0.186 0.814 0]}

The sum of two intermediate outputs (Equation 3) is the predicted fuzzy behavior of *G3 *for the three time points, which can be defuzzified using the point-centroid method (Equation 2) and transformed back to real numbers on [-1,1]:

*G*3 = {[0 1.019 0.981] [0 2.0 0] [0.981 1.019 0]} = {0.491 0 -0.491}

These numbers can be transformed back to a Log2 expression ratio by inverting the normalization (multiplying by π/2 and taking the tangent):

*G*3 = {0.97 0 -0.97}

Finally, we use Equation 4 and the experimental data for *G3 *to calculate the fit error for this rule combination:


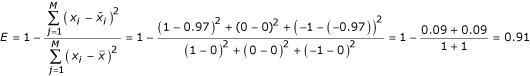


which compares to a maximum *E *= 1.0 for a perfect fit.

### Exhaustive network search

In general, a possible model for a node can include any combination of the genes available to act as inputs. In the work described here, we consider potential interactions of 12 genes. Thus, a rule for any one gene can include as inputs any combination of any number of up to all 11 other genes. Since each input gene can influence the node by any one of the 27 possible fuzzy rules, there are approximately 10^16 ^possible rule combinations for each of the 12 genes, making the exhaustive search method practically impossible. Thus, the number of possible inputs to a node must have a maximum constraint to make exhaustive search tractable.

Studies of network topology through the experimentally observed association of proteins suggest that in many cases only few regulatory proteins are observed to directly influence the expression of a gene [26,30-32]. For our transcriptional network searches, we use the constraint of up to 4 input genes to any node. Thus, for each node gene, each of the other 11 genes occurs as an input alone and also in combination with any of up to 3 of the other genes as multiple inputs. Our use of this input constraint does not necessarily restrict the full range of interactions that can be found for the genes in our network, since *all *possible combinations of 1 through 4 of the genes are searched sequentially. For example, in our fitting of rules to *CLN3*, we considered the following potential input combinations: *SIC1 *alone, *SIC1 *and *CLN1 *together, *SIC1*-*CLN1-CLN2*, *SIC1-CLN1-CLN2-CLN3*, *CLN1*, *CLN1-CLN2*, *CLN1-CLN2-CLN3*, *CLN1-CLN2-CLN3-SWI4*, *CLN2*, *CLN2-CLN3*, etc. If we include all combinations from 1 through 4 of the genes taken from the 11 total possible inputs, then the total search space for each of the 12 genes consists of approximately 10^8 ^rules (taking about 10 minutes on a PowerMac G4 using a single 450 MHz processor). Simulation files used to generate all the data presented here are available from the authors upon request.

## Authors' contributions

BAS originated the concept of applying scalable (URC) fuzzy logic to modeling biological systems, implemented the scheme described within on the data set, and was the primary author. JPF conceived of the approach to use exhaustive search for biological network reconstruction. JNQ and AAQ developed and initially implemented the method of combinatorial input selection for the exhaustive network search, and AAQ contributed to the text. All authors read and approved the final manuscript.

## Supplementary Material

Additional File 1 Microsoft Excel spreadsheets of simulation results. See descriptive text in the workbook. Click here for file

## Figures and Tables

**Table 1:** List of Genes in the Fuzzy Network Model Subset of genes involved in the yeast cell cycle selected for our demonstration network. Descriptions taken from the Yeast Protein Database (Costanzo *et al*., 2000; URL: ).

**GENE NAME**	**ORF**	**DESCRIPTION**
SIC1	YLR079W	inhibitor of the Cdc28-Clb protein kinase complex
CLN1	YMR199W	G1/S-specific cyclin
CLN2	YPL256C	G1/S-specific cyclin
CLN3	YAL040C	G1/S-specific cyclin
SWI4	YER111C	transcription factor, subunit of the SBF factor
SWI6	YLR182W	transcription factor, subunit of SBF and MBF (check this)
CLB5	YPR120C	B-type cyclin
CLB6	YGR109C	B-type cyclin
CDC6	YJL194W	initiates DNA replication, active late G1/S
CDC20	YGL116W	cell division control protein
CDC28	YBR160W	cyclin-dependent protein kinase
MBP1	YDL056W	transcription factor, subunit of MBF

**Table 2:** Best Fitting Rules for *CLB5 *Each row represents rules relating inputs (in columns) to *CLB5*. Rule format described in Methods; e.g., [3 3 1] for *CDC20 *means "If *CDC20 *is **1 **(Low) Then *CLB5 *is **3 **(High); if *CDC20 *is **2 **(Med) then *CLB5 *is **3 **(High); if *CDC20 *is **3 **(High) then *CLB5 *is **1 **(Low)."

**Fit(E)**	**SIC1**	**CDC20**	**CLN3**	**SWI6**	**CLN1**	**CLN2**	**CLB6**	**SWI4**	**CDC28**	**MBP1**	**CDC6**
0.881	---	3 3 1	---	---	---	1 1 3	1 2 3	---	---	---	1 2 3
0.877	---	2 3 1	---	---	---	1 1 3	1 2 3	---	---	---	2 2 3
0.875	2 2 3	2 3 1	---	---	---	1 1 3	1 2 3	---	---	---	---
0.871	---	3 2 1	---	---	---	1 1 3	1 3 3	---	---	---	1 2 3
0.869	---	3 2 1	---	---	---	1 2 3	1 2 3	---	---	---	1 2 3
0.862	---	3 3 1	---	---	---	1 2 3	1 2 3	---	---	---	1 1 3
0.862	---	3 3 1	---	---	---	1 1 3	1 3 3	---	---	---	1 1 3
0.860	---	3 3 2	---	---	---	1 2 3	1 2 3	---	---	---	1 1 3
0.859	---	3 3 2	---	---	---	1 2 3	1 2 3	---	---	---	1 1 2
0.859	---	2 2 1	---	---	---	1 2 3	1 2 3	---	---	---	2 2 3
0.857	---	2 3 1	---	---	---	1 2 3	1 2 3	---	---	---	2 1 3
0.856	---	2 3 1	---	---	---	1 1 3	1 2 3	---	---	---	1 2 3
0.856	---	3 2 1	---	---	---	1 1 3	1 2 3	---	---	---	1 3 3
0.854	2 2 3	3 3 1	---	---	---	1 1 3	1 2 2	---	---	---	---
0.854	1 1 2	3 3 1	---	---	---	1 1 3	2 3 3	---	---	---	---
0.852	2 2 3	2 3 1	---	---	1 2 3	1 1 3	---	---	---	---	---
0.852	---	3 3 1	---	---	---	1 1 3	2 3 3	---	---	---	1 1 3
0.851	---	3 3 1	---	---	---	1 1 3	1 2 2	---	---	---	1 2 3

**Table 3:** Three Best-Fitting Models for Each Gene* Rules are in the same format as in Table 2 (see Methods). In this case, the genes in rows are inputs to genes in columns. The three best combinations of rules are in consecutive rows for each input; i.e., the best fit rule combination is the first row of each input block with the fit score (*E*) in the first row of the last block in the table. * Models are in rows, e.g., the best-fitting model for *SIC1* has the input *CLB5* with the rule (1 3 3), *MBP1* with rule (3 1 1), and *CDC6* with rule (1 2 3) corresponding to the fit error *E *= 0.689.

	*OUTPUTS*											
***INPUTS***	**SIC1**	**CLB5**	**CDC20**	**CLN3**	**SWI6**	**CLN1**	**CLN2**	**CLB6**	**SWI4**	**CDC28**	**MBP1**	**CDC6**
**SIC1**	---								2 2 3		3 2 1	1 1 3
	---	2 2 3	2 1 3					1 3 3			3 2 1	1 2 3
	---	2 2 3	2 1 3					1 3 3			3 2 1	1 2 3
**CLB5**	1 3 3	---						1 3 3				
	1 3 3	---				1 1 3						
	1 3 3	---					1 1 3		3 2 1			
**CDC20**		3 3 1	---	3 3 2		3 2 1						1 3 3
		2 3 1	---			3 3 1						
		2 3 1	---			3 1 1						1 3 3
**CLN3**			3 1 2	---	3 3 1				2 2 3	3 3 2		1 2 3
				---	3 2 1				2 1 3			1 1 3
	3 1 3			---	3 3 1							1 1 3
**SWI6**			1 3 1	3 2 1	---						3 1 2	
**(SBF)**			1 3 1	3 1 1	---				1 2 1		3 1 2	2 3 1
			1 3 1	3 2 1	---					1 2 3	3 1 1	
**CLN1**			3 3 1			---	1 2 3	1 2 3	1 1 2	3 1 1		
			3 2 1			---	1 3 3	1 2 3		3 1 1		
		1 2 3	3 1 1			---	1 3 3	1 3 3		3 1 2		
**CLN2**		1 1 3	3 1 1	3 1 3		1 3 3	---	1 1 3				
		1 1 3	3 2 1	3 1 3		1 3 3	---	1 1 3				
		1 1 3		3 1 3		1 3 3	---	1 1 3				
**CLB6**		1 2 3			1 1 3	1 1 3		---		1 1 3	1 3 3	3 1 3
		1 2 3			1 1 3	1 1 3	1 1 3	---		1 1 3	1 3 3	
			3 3 1		1 1 3	1 1 3		---	1 2 3		1 3 3	
**SWI4**									---		3 2 1	
**(SBF)**	1 1 3			2 3 3					---	3 3 2		
									---		3 2 1	1 2 3
**CDC28**					3 1 3					---		
										---		
					3 1 2					---		
**MBP1**	3 1 1				3 3 1			2 1 3	3 3 1		---	
**(MBF)**	3 1 1				2 3 1			1 2 3	3 3 1		---	
	3 2 1				3 3 1	2 3 2		1 1 3	3 3 1	2 2 3	---	
**CDC6**	1 2 3	1 2 3		1 1 3						3 2 3		---
	1 3 3			1 2 3	1 3 1				3 2 3	3 2 3		---
	1 2 3			2 2 3					2 1 3	3 2 3		---
**Fit (*E*)**	0.689	0.881	0.703	0.792	0.620	0.943	0.877	0.714	0.782	0.510	0.624	0.764
	0.643	0.875	0.702	0.768	0.599	0.930	0.837	0.706	0.768	0.508	0.624	0.758
	0.631	0.852	0.695	0.746	0.596	0.916	0.835	0.705	0.763	0.498	0.622	0.720
